# Sonographically controlled minimally-invasive A1 pulley release using a new guide instrument – a case series of 106 procedures in 64 patients

**DOI:** 10.1186/s12891-023-06982-x

**Published:** 2023-11-10

**Authors:** Damian Sutter, Aline Treier, Esther Vögelin

**Affiliations:** Department of Hand Surgery, University Hospital Berne, Inselspital Bern, Freiburgstrasse 10, CH-3010 Berne, Switzerland

**Keywords:** Hand surgery, Trigger finger, Stenosing tenosynovitis, Ultrasonography, Complications

## Abstract

**Background:**

With percutaneous and minimally-invasive pulley release becoming more popular, safety and reliability of such minimally-invasive procedures remain a concern. Visualization of the technical steps by ultrasound suggests increased safety but shows the potential for harm to tendons, nerves and vessels without proper instrumentation. We present the results of implementing a sonographically guided minimally-invasive procedure in 106 trigger digits of 64 patients between 2018–2021.

**Methods:**

A guide instrument for use with a commercially available hook knife was developed and tested in 16 cadaver hands. Due to complication early in our clinical series this guide was modified in due course. A revised design of the guide has been in use since November 2019 with improved performance and safety.

**Results:**

One hundred six procedures in 64 patients were performed. After guide revision, we report a success rate of 97.3%. Complications after instrument revision include two cases of incomplete pulley release and one case of inadvertent skin laceration. The majority of patients report returning to all strenuous activities within two weeks at most apart from four individuals with prolonged postoperative discomfort.

**Conclusion:**

We present the results of the development and implementation of a novel guide instrument for use with a hook knife to treat trigger finger. Despite several limitations of this study, we show that sonographically controlled, minimally-invasive A1 pulley release can be performed safely and effectively with appropriate surgical instruments and practice.

## Background

Trigger finger is one of the most common diagnoses in hand surgery with an estimated lifetime risk of 2 to 3% [1–3]. Short-term improvement, and sometimes sustained relief of symptoms, may be achieved by infiltration of corticosteroids [4, 5], with a reported response rate between 45 and 80% [6]. The definitive cure of this pathology is achieved by surgical release of the A1 pulley [7]. The open procedure is simple and usually of short duration, performed under local anesthesia in an ambulatory setting, and remains the most widely used surgical treatment for trigger finger. However, some patients suffer from prolonged postoperative discomfort, pain and stiffness, mostly attributed to local edema and scar formation [7–9]. A correlation between longstanding preoperative symptoms and persistent postoperative discomfort has been shown [8, 9]. In our experience, Dupuytren tissue of the palmar aponeurosis present at time of open trigger finger release may be worsened by surgery in the already transformed aponeurosis. A recent study has found higher odds and expedited rate of developing new-onset Dupuytren’s disease after surgery for trigger finger compared to steroid injection for the treatment of trigger finger [10].

Over the past two decades, percutaneous A1 pulley release has been popularized, promising less postoperative swelling and scar formation in the palm as well as faster rehabilitation time and potentially cheaper procedure [11–14]. Although blind percutaneous release has been propagated by numerous authors [11, 12, 15], sonographically controlled percutaneous release has been shown to increase success and reduce complication rate and postoperative pain [16–20]. Another advantage of percutaneous or minimally invasive release is the negligible or small incision through the palmar aponeurosis avoiding irritation of the palmar aponeurosis. Moreover, when compared to open pulley release, return to work was shown to be quicker and patient satisfaction with regards to the cosmetic appearance of the scar seems higher in sonographically controlled percutaneous and minimally invasive procedures [21]. Cadaveric studies [3, 22–26] have examined the feasibility of different techniques. A combination of percutaneous release with injection of corticosteroids during seems to be advantageous, leading to reduced swelling after the procedure without in the risk of increased wound healing complications [27, 28].

Before first clinical use of minimally-invasive trigger finger release, the technique was tested in a cadaver setting. Several decisions were made based on the anatomic work of Rojo-Manaute et al. [23, 29, 30], who have described a safe area to perform the release from an intrasheath position, rather than an extrasheath position. It was shown that the technique can be safely performed in all fingers except for the thumb. Chern et al. [13, 14, 22] were among the first authors who described release of the A1 pulley with a hook knife, albeit in an extrasheath position. Before the introduction of the knife, a blunt probe was inserted, which was used as a dilator, causing less injury to adjacent structures [13].

In this study, we aim to combine the use of a hook knife with the intrasheath position.

We performed ultrasound-guided minimally-invasive A1 pulley release in 16 cadaver hands using a hook knife in the intrasheath position. Placement of the hook within the sheath proved difficult, retraction from an unfavorable position unlikely without damage to the flexor tendons. Therefore a guide instrument was developed for ease of hook knife insertion and flexor tendon protection. The technique was subsequently applied in clinical use and the instruments were further refined in due course.

## Materials and methods

### Cadaver experiments

This study received approval from our Ethics Committee (Kantonale Ethikkommission BE 2018–00264) and was performed in 2018.

Sixteen cadaver hands were available for use in this study. To focus on the operative technique, only index through small fingers were treated. After each attempt, the pulley, tendons and neurovascular structures were dissected and inspected to assess for success of pulley release and possible injuries to adjacent structures. Pulley release was performed with a commercially available hook knife (trigger finger retrograde knife, Smith & Nephew, Inc., Memphis, TN, USA) The use of this instrument alone did not allow for intrasheath placement without the risk of damage to the flexor tendons. Additionally, insertion into the intrasheath position proved to be difficult due to the blunt tip of the knife. Therefore, a guide instrument was designed with the specific purpose to open the flexor tendon sheath, facilitate insertion and to protect the flexor tendons during placement of the hook knife. It would also allow retraction of the hook knife in case of poor initial placement. A number of different guide instrument designs were assessed in cadavers. The addition of a channel to guide the hook knife improved surgical accuracy and ease of knife placement. The use of this instrument allowed successful release in all fingers in the cadaver setting. The surgical technique is described below.

### Retrospective case series

We conducted a retrospective case review of 106 sonographically guided A1 pulley release procedures in a total of 77 operations in 64 patients from November 2018 until September 2021. We included all patients with a clinically diagnosed trigger digit of grade Green II-IV [31] who were operated with the minimally-invasive technique who have signed a written consent form. The exclusion criteria were age under 18 and lack of general consent status. All procedures were performed as an ambulatory procedure under local anesthesia by two surgeons [DS, EV].

The hook knife and our guide instrument were used. To improve the technique, two different guide instrument designs were used, changing from one to the other in November 2019. Procedures were performed in adherence to standard surgical sterility, using standard surgical dressings in an operating theater. The intraoperative sonographic visualization and postoperative examination were performed using a Philips Epiq 5G Medical system (Philips, Bothell, WA, USA) with a 17.5 MHz hockey stick probe which was covered in a single-use sterile dressing.

We explored the feasibility of utilizing sonography as a diagnostic test for assessing complete A1 pulley release, a measurement that has not been established thus far. Specifically, the cross-sectional area (CSA) of the flexor tendons in an axial view at the same defined level of the MCP joint in full finger extension as well as full active PIP and DIP flexion was measured before and after pulley release and their relative change calculated. Additionally, the complete release of the A1 pulley was dynamically tested with a surgical dissector. Finally, clinical lack of triggering was confirmed.

### Surgical technique

**Fig. 1:**
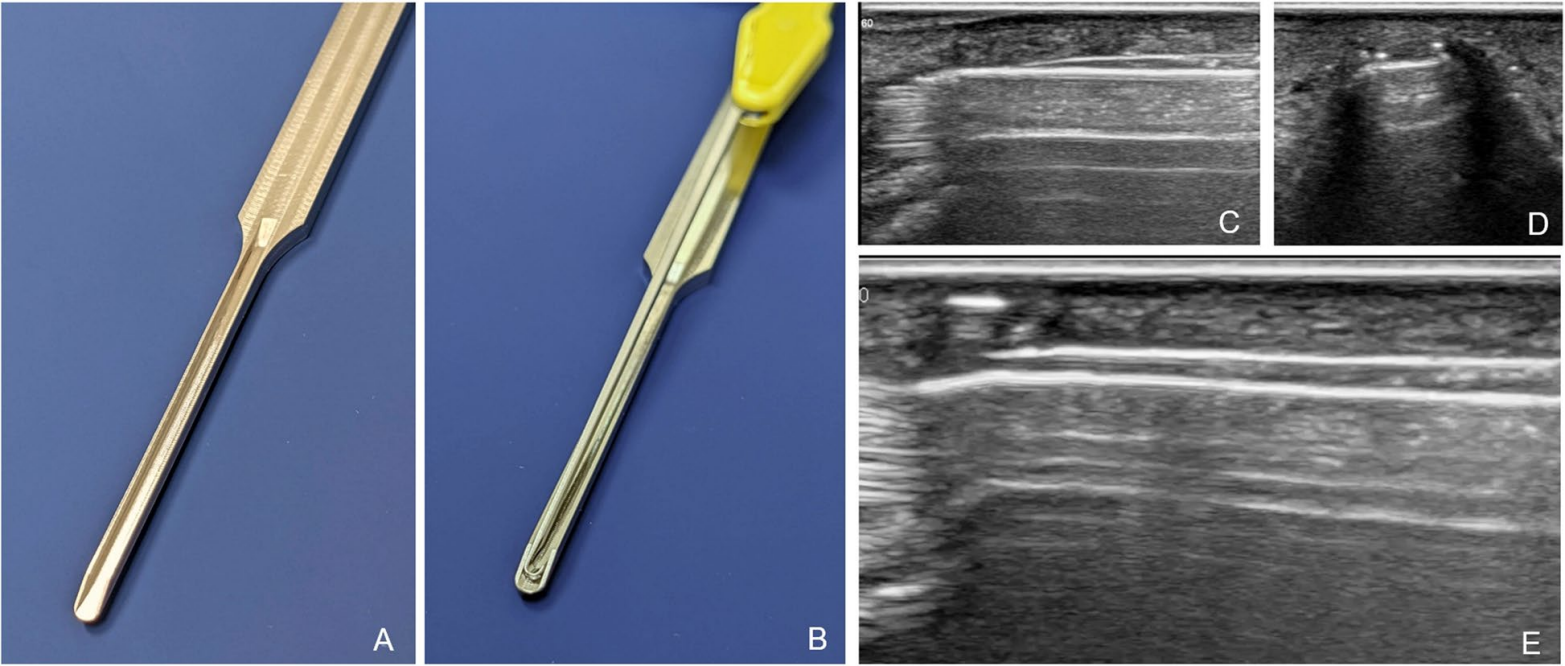
**A**-**E** Guide instrument and hook knife with sonographic view. **A** Guide instrument, as of November 2019; **B** guide instrument shown with inserted hook knife; **C** longitudinal and **D** axial sonographic view of guide instrument in the intrasheath position; **E** hook knife placed upright within channel just proximal to the A1 pulley

The procedure is performed in WALANT (wide awake local anesthesia no tourniquet technique, mepivacaine 1%, epinephrine 1:100 000, sodium bicarbonate 8.4% 1:10) using 4-6 ml per finger injected into the palm of the involved digit. The hand is placed palm up, with MCP joints hyperextended over a roll of surgical towels. A 3–4 mm wide transverse incision is placed 3–4 mm distal to the palmar-digital crease over the proximal phalanx. Scissors are used to dissect the subcutaneous tissue away from the flexor tendon sheath. The A1 pulley is then sonographically visualized and the guide instrument is inserted into the flexor tendon sheath just distal to the A1 pulley and advanced proximally between the A1 pulley and the flexor tendons. Once placed at the proximal edge of the A1 pulley, central location of the guide instrument palmar to the flexor tendon is confirmed in an axial view. The hook knife is inserted flat through the channel in the guide instrument (Fig. 1A – E). Its passage to the proximal edge of the A1 pulley can easily be visualized. The central position over the pulley is affirmed once again before the hook knife is turned 90° and by retraction of the hook knife distally the pulley is released. After retraction of the hook knife, a blunt probe, such as a surgical dissector, is inserted into the flexor tendon sheath to feel for residual fibers. This can be considered both a clinical and sonographic test.

Two different versions of the guide instruments were in use, with a design revision in November 2019. The first design acted as a dilator of the intrasheath space. A slot facing the the flexor tendons allowed for insertion of the hook knife without injury to the flexor tendons. However, in order to release the pulley, the guide instrument had to be retracted first. The revised guide instrument has a slot facing the pulley and can therefore be left in place for the duration of the procedure, thus continually protecting the flexor tendons and further simplifying the procedure.

Patients were followed up in our outpatient clinic within 1–2 days postoperatively, as well as 2 and 6 weeks postoperatively. Further follow-up consultations were planned as needed. All Patients were contacted by phone between 9 and 12 months postoperatively. Routinely, patients were seen by our hand therapists once for instruction. If flexion or extension of the operated digit were deemed unsatisfactory at any given follow up time point, additional sessions with a hand therapist were scheduled. Postoperative pain management included paracetamol and NSAID.

## Results

### Cadaver study

Minimally-invasive A1 pulley release was performed on a total of 16 cadaver hands, focusing on index, long, ring and small fingers (*n* = 64). Early experience was gained with the use of the hook knife alone (*n* = 4). The need for a guide instrument, aiding in the placement of the hook knife in the intrasheath position was obvious. The use of different dilators and early versions of a guide instrument with a channel accounted for 42 pulleys, of which 32 procedures were successful. No injuries to nerves or vessels were observed, but in 8 cases injury to the superficial flexor tendons occurred. The final instrument design was applied in the remaining 18 pulleys where we achieved a 100% success rate and we noted no signs of injuries to the tendons or neurovascular structures. This instrument was chosen to be used in our early clinical experience.

### Case series

Sixty-four patients operated between November 2018 and September 2021 were included in this review. 39 women (60.9%) and 25 men, mean age 62.7 (range 37–88). Mean time of onset of symptoms prior to the operation was over one year, ranging from a few weeks, to several years (mean 57.8 weeks, median 52 weeks). 64 of 106 digits had previously been treated with a cortisone injections within 2 years of the operation either in our clinic or by the family physician. Significant comorbidities such as type I or type II diabetes, rheumatoid diseases, dupuytren’s disease, advanced cardial and renal insufficiency were present in 41 patients.

Seventy-seven procedures accounted for 6 thumbs, 14 index, 42 long, 32 ring and 12 small fingers. 5 female and 13 male patients had two or more digits addressed simultaneously, two individuals had four digits treated at the same time. In 17 patients simultaneous carpal tunnel release was performed.

Until October 2019, 7 complications were observed in a total of 31 procedures in 26 patients (21.8%). Conversion to open release had to be performed on 8 occasions (all in middle and ring fingers) due to an unfavorable position of the hook knife. In all 8 cases, we inspected the flexor tendons and the neurovascular bundles. In 5 cases, partial lesions to the superficial flexor tendons were found (5–20% affected cross-sectional area) and debrided. There weren’t any injuries to nerves or vessels. Postoperative recuperation was uneventful and postoperative pain was not increased in these 5 cases. The remaining two complications were postoperative infections. These cases presented as follows:

The first patient was a 69-year-old lady with a grade III trigger finger of her middle and ring finger. Steroid infiltration 2.5 months before surgery was ineffective. Intraoperatively, placement of the hook knife was difficult and residual fibers had to be addressed in her long finger, whereas the release of the A1 pulley in the ring finger was comparatively simple. A follow-up visit after 12 days showed no sign of infection and the patient reported satisfactory results. Sonographically, some peritendinous fluid was visible. The patient reported increased swelling of the long finger only after three weeks. She was subsequently operated on. Intraoperative findings showed some turbid fluid, but no pus. The pulley was shown to be fully released. The fourth ray remained free of infection. Swabs were positive for Pseudomonas aeruginosa and the patient was treated with antibiotics accordingly. In due course, flexion deficit of the long finger remained present with sonographically present bow stringing due to an insufficient A2 pulley, likely secondary to the infection. The patient opted against revision surgery despite residual impaired function.

The second patient was a 74-year-old gentleman with a grade II trigger finger of the little finger. Intraoperatively, placement of the probe proved to be somewhat difficult, but safe placement of the hook knife was achieved on a second attempt. Otherwise, the surgery was uneventful. The patient reported swelling since day 8 after surgery and finally presented on day 11. Surgical revision showed putrid flexor tenosynovitis. A Staphylococcus aureus infection was treated with a 14-day course of antibiotics. Two weeks after revision surgery, the patient was pain-free with intact sensibility and full range of motion. 1 year later the patient reported unimpaired function.

The occurrence of these complications has resulted in the revision of the guide instrument after 31 procedures. It was redesigned to improve ease of insertion of both the guide instrument and the hook knife and to increase protection of the flexor tendons. Since the implementation of the revised guide in November 2019, conversion to an open procedure was necessary twice (2.6%). Once due to an unsafe position in a thumb, once in a patient who had adverse effects to the local anesthesia. In 75 procedures between November 2019 and September 2021, we have two complications to report (2.6%): A skin laceration over the A1 pulley occurred when residual fibers were addressed with the hook knife. The laceration healed without sequelae. The other patient had residual pulley fibers, likely an A0 pulley, resulting in occasional residual triggering. The fibers were operated once again after 11 months using our technique leading to complete and lasting resolution of the issue. In summary, overall success rate after implementation of the revised guide instrument was 97.3%, with a conversion rate of 2.6%. No persistent or recurrent triggering was observed except in one young manual worker with two simultaneous trigger fingers, one of which had to be operated again after 11 months using the same technique.

**Fig. 2:**
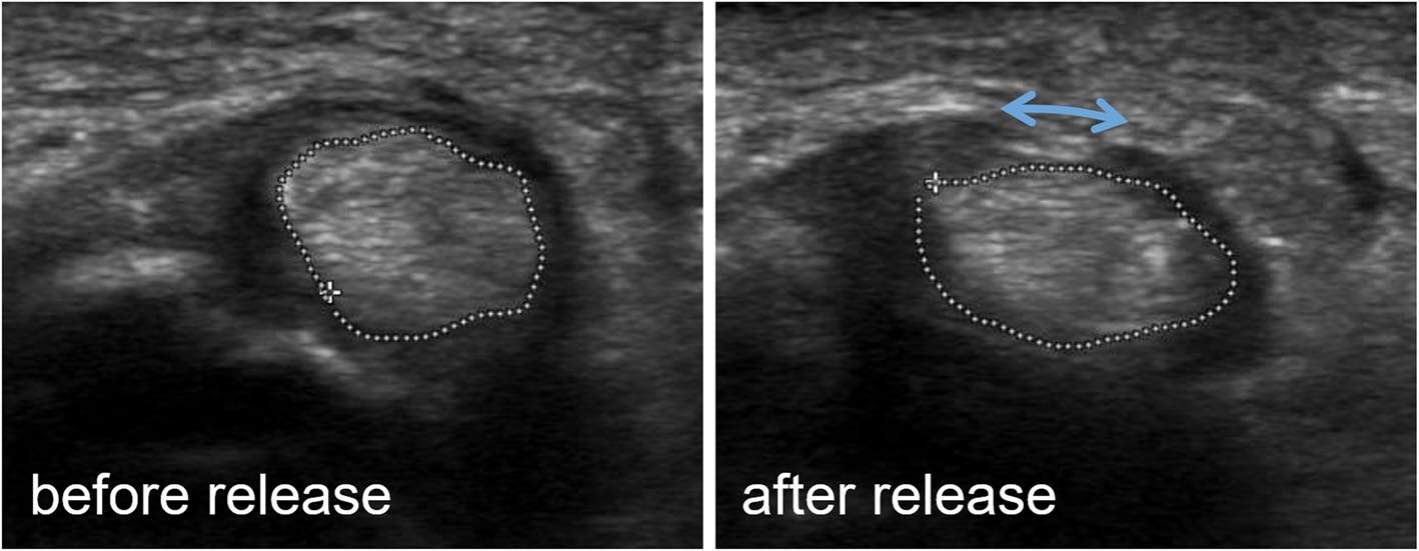
cross-sectional area of flexor tendons before and after pulley release, retraction of sectioned pulley visible on flexion after pulley release

**Fig. 3:**
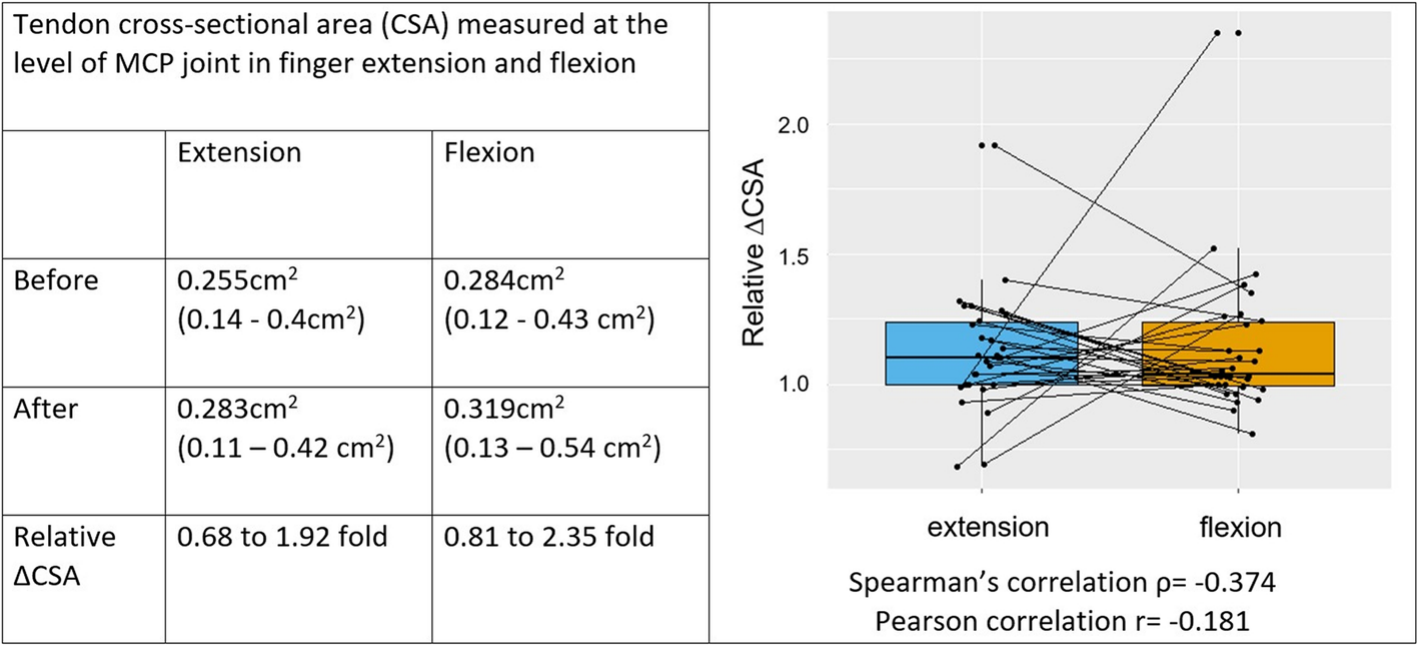
Correlation analysis of relative change of cross-sectional area in extension and flexion of the finger before and after pulley release. Matched pair analysis is shown in a boxplot diagram

CSA measurements of the flexor tendons were performed on sonographic images before and after pulley release (Fig. 2). Matched and paired sonographic images were analyzed in 30 early cases. Measured CSA varied greatly and matched pairs correlated poorly. Results are summarized in Fig. 3.

**Fig. 4:**
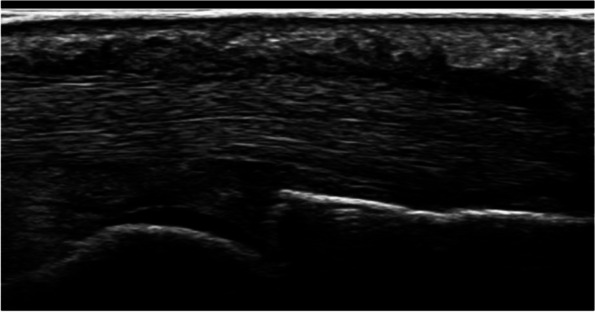
postoperative subcutaneous hypoechogenic signal

Sonographic findings at postoperative follow-up consultations revealed persistent peritendinous and subcutaneous hypoechogenic area in 26 patients, not including the two cases with postoperative infections (Fig. 4). Of these patients, most were symptom-free by that point. On average these sonographic findings were present up to 6 weeks postoperatively, though follow-up for many patients ended by that point, as they were asymptomatic. Of note is one patient with persistent subcutaneous edema up to 6 months postoperatively upon opportunistic sonographic assessment despite being asymptomatic since 14 days after the operation—the patient in question has later been diagnosed with Sjögren’s syndrome. Only 4 patients reported residual pain beyond 6 weeks postoperatively. Return to daily activities and pain relief in these four cases were noted at follow-up visits between 3 and 6 months postoperatively.

All patients were asked about the postoperative course at least 9 months after the intervention by a telephone call, unless they were seen in our clinic by that time.

## Discussion

While open A1 pulley release is attributed to a high success rate nearing 100% and is only of short duration, postoperative pain and discomfort are a frequent occurence. A large multicenter cohort study of 1879 patients recently published a total complication rate of open A1 pulley release of 17.1%, with 7% needing additional analgesia and hand therapy, 7.8% requiring steroid injection or antibiotics and 2.1% requiring surgical revision [32]. Longstanding preoperative symptoms are attributed to prolonged postoperative symptoms [8, 9]. Another reason for postoperative discomfort has been attributed to the disturbance of palmar aponeurosis and subcutaneous tissue and to the scar formation [7, 33]. Both diabetes and multiple digit surgery have both been associated with an elevated risk of postoperative infection [34].

Over the past ten years, percutaneous and minimally-invasive A1 pulley release procedures have become more prevalent. Initially blind percutaneous procedures were performed, but studies have shown a high risk of digital nerve injury and a higher risk of residual triggering when compared to sonographically controlled percutaneous pulley release [18, 20, 35]. Sonography has repeatedly been shown to be beneficial regarding outcome and patient satisfaction since [21].

To our knowledge, no authors have macroscopically investigated for possible damage to the flexor tendons that may lead to postoperative discomfort or even triggering after pulley release when using a percutaneous needle technique.

Position of the cutting device relative to the flexor tendon sheath has been studied in cadavers. An intrasheath position has the lowest risk to the digital nerves [29, 30]. The use of a hook knife has previously been described in an extrasheath position [14], but not an intrasheath position. With our experience in cadaver hands we have learned the intrasheath position of a hook knife to risk injury to the flexor tendons, which is why we developed an instrument to guide the hook knife to a safe position.

Our cadaver study demonstrates an alternative, safe method of a sonographically controlled, minimally-invasive A1 pulley release using a hook knife with a distal to proximal insertion in an intrasheath position with the use of a protective guide instrument.

In our case series, unfavorable position of the hook knife occurred in 8 cases prompting a revision of the guide instrument design. Safe and easy placement of a hook knife within the guide instrument has since been achieved readily and consistently since design revision. The intrasheath position, as suggested by Rojo-Manuate [29, 30], proved to be effective and safe in conjunction with our revised guide instrument. The technique can be performed in all fingers including the thumb, but we suggest being cautious and converting the surgery to an open procedure if sonographic visualization is less than optimal, but certainly if the hook knife is in an unfavorable position within the substance of the flexor tendons. This may be the case if the MCP joint cannot be passively extended which is a common problem for the thumb. Extra care must be taken to visualize the radial digital nerve of the thumb, which is crossing the flexor tendon just proximal to the A1 pulley [15, 36].

Early in our series, using the first instrument design, two major complications arose. Two cases of infections were treated accordingly. One patient has had a full recovery and has since returned for minimally-invasive release of a pulley on the other hand. The other patient has some residually limited range of motion, but barely feels restricted in her daily activities. Whether the cortisone injection 2.5 months before surgery is partially responsible is unclear, but a recent study suggests avoiding pulley release in patients within 31 to 90 days post-injection [37]. Interestingly, it has been shown that with combined corticosteroid injection during percutaneous release, postoperative swelling has been reduced without wound healing complications [27, 28].

Other complications were only visible in cases that needed conversion to an open procedure and were caused by incorrect placement of the guide instrument or hook knife. After design revision, conversion to an open procedure has been necessary twice as a precautionary measure and we have not seen injuries in these two cases. The current design of the guide instrument has never entangled the flexor tendons and makes it near impossible for the hook knife to be placed in an unfavorable position. The surgical technique was thus simplified and can easily be performed in all grades of stenosing tenosynovitis.

An attempt to sonographically assess complete pulley release was made. CSA of the flexor tendons at the level of the MCP joint in extension and flexion before and after pulley release was measured and compared but proved inconclusive and unpractical. However, we have found the use of a blunt or hooked probe inserted via the guide instrument to be a simple and reliable method to test for residual pulley fibers, both visually and tactile. If residual fibers are suspected, specific release with the hook knife should be performed carefully. No recurrence or persistent triggering was observed within 6 weeks after treatment except in one patient with recurrence due to an A0 pulley after 6 weeks. He was successfully reoperated with the same technique after 11 months.

Of note is an increase of postoperative hypoechogenic subcutaneous tissue up to 6 weeks postoperatively, observed to varying extent in 26 digits (24.5%). A similar finding has been described by Chopin et al. [38], but in a lower percentage of patients (5.7%). It is unclear whether this sonographic finding may be considered normal 6 weeks postoperatively or if it truly represents a higher level of inflammation. Contrary to open pulley release, synovectomy is not possible in percutaneous and minimally-invasive surgery and may account for this sonographic finding present up to several months postoperatively. Several authors describe the use of concurrent cortisone injection, which may address this issue [27, 28, 38, 39]. However, the vast majority of patients in our small case series were asymptomatic despite the presence of this sonographic finding. We do not have a control cohort treated with open pulley release and can therefore not comparatively report on postoperative sonographic findings.

This study includes the experience of development and first implementation of a novel technique at our institution, which comes with a steep learning curve. We recognize the severe limitations to this retrospective patient chart review. Comparative findings, such as postoperative pain levels, strength, and range of motion were documented inconsistently and therefore do not allowing analysis. We also lack patient-reported outcome scores. Ideally, a prospective study with open pulley release as a control group could highlight the potential advantages of one technique over the other.

## Conclusion

Sonographically assisted A1 pulley release allows for real-time transcutaneous intraoperative monitoring of percutaneous and minimally-invasive procedures such as A1 pulley release. A blunt guide instrument has been developed to facilitate the intrasheath placement of a hook knife. The procedure has a steep learning curve and an early version of the guide instrument was unable to consistently guide the hook knife to a safe position. After the introduction of a revised guide we’ve seen a high success rate at 97.3%, with a low conversion and complication rate at 2.6% each. The lack of patient reported outcome scores and inconsistent documentation limit the value of our case series. However, they represent the daily clinical setting we all face and also highlight the difficulties in the introduction of a novel surgical technique. Despite these limitation, we regard this technique as potentially beneficial in several cases, most notably when addressing multiple digits at once, especially in manual workers. Scar formation is minimal, which may cause less irritation of the palmar aponeurosis and pose less of a risk of Dupuytren’s disease progression or activation or the formation of Dupuytren’s disease.

## Data Availability

The datasets used and analyzed during the current study are available from the corresponding author on reasonable request.

## Abbreviation

CSA: Cross-sectional area
